# The Wnt Signaling Pathway Effector TCF7L2 Mediates Olanzapine-Induced Weight Gain and Insulin Resistance

**DOI:** 10.3389/fphar.2018.00379

**Published:** 2018-04-16

**Authors:** Ranran Li, Jianjun Ou, Li Li, Ye Yang, Jingping Zhao, Renrong Wu

**Affiliations:** ^1^Department of Psychiatry, The Second Xiangya Hospital of Central South University, Changsha, China; ^2^Shanghai Institute for Biological Science, Chinese Academy of Sciences, Shanghai, China

**Keywords:** olanzapine, Wnt signaling pathway, atypical antipsychotics, TCF7L2, weight gain, insulin resistance

## Abstract

Olanzapine is a widely used atypical antipsychotic medication for treatment of schizophrenia and is often associated with serious metabolic abnormalities including weight gain and impaired glucose tolerance. These metabolic side effects are severe clinical problems but the underpinning mechanism remains poorly understood. Recently, growing evidence suggests that Wnt signaling pathway has a critical role in the pathogenesis of schizophrenia and molecular cascades of antipsychotics action, of which Wnt signaling pathway key effector TCF7L2 is strongly associated with glucose homeostasis. In this study, we aim to explore the characteristics of metabolic disturbance induced by olanzapine and to elucidate the role of TCF7L2 in this process. C57BL/6 mice were subject to olanzapine (4 mg/kg/day), or olanzapine plus metformin (150 mg/kg/day), or saline, respectively, for 8 weeks. Metabolic indices and TCF7L2 expression levels in liver, skeletal muscle, adipose, and pancreatic tissues were closely monitored. Olanzapine challenge induced remarkably increased body weight, fasting insulin, homeostasis model assessment-insulin resistance index, and TCF7L2 protein expression in liver, skeletal muscle, and adipose tissues. Notably, these effects could be effectively ameliorated by metformin. In addition, we found that olanzapine-induced body weight gain and insulin resistance actively influence the expression of TCF7L2 in liver and skeletal muscle, and elevated level of insulin determines the increased expression of TCF7L2 in adipose tissue. Our results demonstrate that TCF7L2 participates in olanzapine-induced metabolic disturbance, which presents a novel mechanism for olanzapine-induced metabolic disturbance and a potential therapeutic target to prevent the associated metabolic side effects.

## Introduction

Schizophrenic patients possess an approximately 20% shortened lifespan compared with the general population. One of the main causes of premature mortality is metabolic syndrome (MetS) (Hennekens et al., 2005; Raedler, 2010), which is twice higher in schizophrenia patients, featuring insulin resistance, glucose intolerance, dyslipidemia, hypertension, type 2 diabetes mellitus (T2DM), cardiovascular disease, and obesity (Rethelyi and Sawalhe, 2011). Largely due to MetS (Ryan et al., 2003; Mathieu et al., 2009; Rheaume et al., 2009), the first-episode, drug-naïve patients present impaired glucose tolerance, insulin resistant, higher levels of plasma glucose (Ryan et al., 2003; Spelman et al., 2007), and increased visceral fat distribution (Thakore et al., 2002; Ryan et al., 2004). In a recent systematic review and meta-analysis (Mitchell et al., 2013b), the overall incidence rate of MetS is 32.5% in schizophrenia patients and related disorders. In clozapine-prescribed patients, the proportion could be as high as 51.9% than that in unmedicated patients (20.2%) (Mitchell et al., 2013a). Furthermore, MetS is also associated with increased risk of cardiovascular diseases and all-cause mortality (Lakka et al., 2002).

Increasing evidence shows that atypical antipsychotics (APPs) are associated with metabolic adverse effects, such as weight gain, obesity, glucose intolerance, dyslipidemia, and MetS (Newcomer, 2007; De Hert et al., 2011; Mitchell et al., 2013b). Compared to the first-episode and unmedicated schizophrenia patients, the prevalence of metabolic disturbance is significantly higher in patients on established antipsychotic drugs (9.8% for unmedicated, 9.9% for first episode, and 35.3% for medicated patients) (Chadda et al., 2013; Mitchell et al., 2013a). Numerous studies have demonstrated that APPs are crucial in the high prevalence of MetS in patients with schizophrenia (Alvarez-Jimenez et al., 2008; Malhotra et al., 2013). Among APPs, olanzapine is widely used for management of patients with schizophrenia and other psychiatric disorders and produces the most serious abnormalities in glucose and lipid metabolism (Alvarez-Jimenez et al., 2008; Komossa et al., 2010). The molecular mechanism underlying olanzapine-induced metabolic disturbance remains largely unknown, although H(1)-histamine receptor has been involved in the APPs-induced weight gain (Kroeze et al., 2003). Interestingly, molecular genetics data show that genes regulating glucose metabolism predispose human population to schizophrenia susceptibility (Hansen et al., 2011; Alkelai et al., 2012). Of these genes, *TCF7L2* is found to be associated with schizophrenia, which is the best replicated risk factor for T2DM, and exhibits the strongest association to diabetes susceptibility (Grant, 2012). Previous study suggested that TCF7L2 may stimulate the pancreatic β-cells proliferation and affect the production of glucagon-like peptide-1 in intestinal endocrine cells (Jin and Liu, 2008). As a transcriptional regulator of the canonical Wnt signaling pathway, it also regulates cell fate specification during development and cell proliferation (Peifer and Polakis, 2000; Clevers, 2006; MacDonald et al., 2009). Previous study suggests that Wnt signaling pathway may be associated with schizophrenia, and expression of Wnt-related proteins is altered following APPs treatment, for example, the expression of β-catenin and glycogen synthase kinase-3 (GSK-3) protein are increased in rat medial prefrontal cortex and striatum after APPs administration (Alimohamad et al., 2005a).

Indeed, converging evidence has recently showed that the protein kinase B (Akt)/GSK-3 and Wnt signaling pathways could play a key role in the pathogenesis of schizophrenia and the molecular mechanisms of APPs (Alimohamad et al., 2005a; Freyberg et al., 2010; Singh, 2013). It has been reported that AKT1 gene polymorphisms are associated with schizophrenia (Xu et al., 2007), and antipsychotic drugs modulate the Akt/GSK-3 and Wnt signaling pathways in order to correct the deficits induced by the gene mutation (Alimohamad et al., 2005b). Furthermore, the downstream molecule of the diabetes risk genes, *TCF7L2*, is associated with schizophrenia (Hansen et al., 2011). These findings prompt us to investigate the possible involvement of the TCF7L2 in olanzapine-induced metabolic disturbances.

Metformin, a widely used biguanide antihyperglycemic drug for T2DM, has been effectively used to prevent antipsychotic-induced weight gain and other metabolic adverse events (Jarskog et al., 2013; Boyda et al., 2014). Metformin normalizes blood glucose levels by suppressing hepatic gluconeogenesis and increases peripheral tissue insulin sensitivity (Kirpichnikov et al., 2002).

In our current study, our goal was to explore the molecular mechanisms and the protective effects of metformin against olanzapine-induced metabolic disturbance. Male mice were included in order to exclude sex differences (Cooper et al., 2007; Wu et al., 2007; Li et al., 2016). Mice were subject to olanzapine, olanzapine plus metformin, or saline for 8 weeks, respectively, and the variables including weight, fasting blood glucose, and insulin and oral glucose tolerance test (OGTT) were determined prior to and after drug administration. Blood lipid profile and the expression of TCF7L2 were also monitored in individual tissues at the end of each treatment paradigm.

## Materials and Methods

### Animals

Male C57BL/6 mice (18.9–22.6 g, 26–30 days old) were obtained from Hunan Slack King Laboratory Animal Co., Ltd. They were housed at 22 ± 2°C, 55 ± 15% humidity on a 12 h light/dark cycle (lights on at 7:00 am). Food and water were allowed *ad libitum* throughout the study. The mice were fasted for about 12 h before the start of experiments (when the mice were 8-week-old). This study was carried out in accordance with the recommendations of Guide for the Care and Use of Laboratory Animal (NRCU, 1996), Animal Ethics Committee of the Second Xiangya Hospital of the Central South University. The protocol was approved by the Ethics Committee of the Second Xiangya Hospital of Central South University. After 1 week of acclimatization, the 8-week-old mice were randomly divided into three groups (10 per group) as follows: group 1 (sham mice) received a standard chow diet plus saline, group 2 received a standard diet plus olanzapine, and group 3 received a standard diet plus olanzapine and metformin.

### Drug Treatment

Olanzapine (brand name: Zyprexa) was purchased from Eli Lilly, United States. Metformin hydrochloride was obtained from Hunan Xiangya Pharmaceutical Co., Ltd., Changsha, China. Olanzapine was dissolved in 0.9% saline solution and maintained in one gavage administration (4 mg/kg/day) every day for 8 weeks. Olanzapine (4 mg/kg/day, oral) and metformin hydrochloride (150 mg/kg/day, oral) were prepared as previously (Matsui et al., 2010; Savoy et al., 2010). The vehicle solution for metformin was 0.9% saline solution. All the drugs were prepared freshly prior to usage and administered orally (gastric gavage) between 9:00 and 14:00 h every day.

### Study Design

Mice (*n* = 10 per group) were randomly assigned into three groups. Group 1 was subject to daily gavage of 0.9% saline solution, while group 2 received daily gavage of olanzapine and group 3 was given olanzapine and metformin. After 1 week of acclimatization and fasting for 12 h, the baseline measurement of body weight, whole-blood glucose level, serum insulin level, and OGTT were determined prior to the administration of olanzapine. The body weight of the mice was monitored weekly. After 8 weeks of treatment, 10 mice in each group were fasted 12 h and gavaged with glucose (2 g/kg body weight), blood glucose was measured at baseline and at 0, 30, 60, 90, and 120 min after glucose load. On the next day, at least 8 h after being fasted, the mice were killed by decapitation. Blood samples were collected, serum insulin level, blood lipid level [including total cholesterol, low-density lipoprotein cholesterol (LDL-C), triglycerides and, high-density lipoprotein cholesterol (HDL-C)], and OGTT were determined. The liver, adipose, skeletal muscle, and pancreatic tissues were collected, immediately frozen in liquid nitrogen, and stored at -80°C until further analysis. A part of the pancreatic tissue was fixed with 4% paraformaldehyde in PBS and stored at 4°C for immunofluorescence staining.

### Metabolic Measures

Blood glucose was determined by clipping tails and using the glucometer (EKF Diagnostics, Germany). For fasting insulin measurement, blood samples were collected and centrifuged (3500 rpm, 20 min, 4°C) to separate the serum and stored at -80°C until assay. Serum insulin level was measured quantitatively using a Mouse Ultrasensitive Insulin ELISA kit (ALPCO Diagnostics, United States). The mice fasted for 12 h were given with glucose (2 g/kg, p.o.). Blood samples were collected from tail tip incision at 0, 30, 60, 90, and 120 min after glucose administration. Blood glucose concentration was plotted against time, and area under the curve (AUCg) was calculated following trapezoidal rules (Dora et al., 2008). Serum concentrations of triglycerides, total cholesterol, HDL, and LDL were measured with an autobiochemical analyzer (C8000, Abbott, United States).

Insulin resistance index was calculated based on the homeostatic model assessment of insulin resistance (HOMA-IR): [fasting insulin (mIU/L) × fasting glucose (mmol/L)]/22.5 (Mather, 2009).

### RNA Extraction and Real-Time Quantitative PCR

Total RNA was extracted from pancreatic tissues by using the SYBR Green PCR kit (F-415XL, Thermo, United States). RNA was reverse-transcribed using the protocol provided in the kit (K1622, Thermo, United States). The primer sequences are listed in **Table 1**. The gene was amplified through RT-PCR method using the SYBRGreen PCR kit (Thermo, United States). GAPDH was used as the reference gene. Amplification was run for 40 cycles. Samples were denatured at 95°C, followed by annealing at 60°C. The mRNA expression of the *TCF7L2* gene was quantitatively analyzed using Applied Biosystems 7300 Real-Time PCR System (Applied Biosystems, Thermo, United States). Data were analyzed with 2^-ΔΔ CT^ (Schmittgen and Livak, 2008).

**Table 1:** PCR primer sequences used to quantify mRNA levels of *TCF7L2* gene by real-time PCR.

Primer	Sequence	Pos	Size (bps)
TCF7L2 F	5′-GTCCTCGCTGGTCAATGAATC-3′	
TCF7L2 R	5′**-**CCGCTTCTTCCAAACTTTCCC-3′	667–791 C	125
GAPDH F	5′-ATCACTGCCACCCAGAAG-3′	
GAPDH R	5′-TCCACGACGGACACATTG-3′	585–775	191

### Immunofluorescence Staining and Imaging

Pancreatic tissues were fixed for 4 h in 4% paraformaldehyde in PBS and embedded for paraffin sectioning (5 μm). The sections were deparaffinised, rehydrated, and incubated overnight at 4°C with goat antisera against insulin, TCF7L2 antibody (1:60–70, D-4, sc-166699, Santa Cruz, CA, United States), and DAPI (AR1176, Wuhan Boster Company). The sections were subsequently probed with secondary antibodies for 20 min at 37°C (Yang et al., 2012). Images of the pancreatic tissues were acquired using a fluorescent inverted microscope (Olympus IX71, Japan). For morphometric analysis, the fluorescence intensity of pancreatic sections was quantified using the Image J 1.37c^1^.

### Western Blot Analysis

TCF7L2 proteins in the pancreas were extracted for Western blot analysis (Yang et al., 2012). Frozen tissues were homogenized in RIPA lysis buffer (Solarbio, Beijing, China) and centrifuged at 12,000 rpm for 10 min at 4°C to collect the supernatant. Protein concentrations of the tissue lysates were determined by bicinchoninic acid method. Tissue lysates were separated by SDS–PAGE and transferred to PVDF membranes. Proteins were probed with rabbit anti-TCF7L2 (1:2500, Abcam Inc., United Kingdom) or mouse anti-actin (1:1000, TA-09, ZSGB-Bio Co., Ltd., Beijing, China) antibodies and incubated with peroxidase-conjugated affiniPure Goat Anti-Mouse IgG (H+L) secondary antibody (1:3000, ZB-2305, ZSGB-Bio Co., Ltd., Beijing, China). The proteins were visualized using a Western Lightning Plus Enhanced Chemiluminescence reagent (ECL, Amersham, United States). Density of the bands was analyzed with a GDS-8000 system (UVP CA, United States).

### Statistical Analysis

Statistics was performed using SPSS 19.0 (Chicago, IL, United States). Statistical differences in measures of the different groups were analyzed by one-way ANOVA followed by Tukey’s multiple-comparison *post hoc* test. The weight levels at different time points were compared across groups using repeated measures ANOVA. Statistical power of the main results was calculated with G^∗^Power 3.1. Correlations were identified using Pearson’s correlation. Multivariate linear regression was performed to examine the relationship between TCF7L2 expression and the change of weight, fasting blood glucose, fasting insulin, AUCg, and HOMA-IR during the 8-week study period. A second analysis was conducted with the metabolic indexes as independent variables and TCF7L2 expression as the dependent variable, with the probability of entry set at 0.10 and removal at 0.15, reporting the coefficient of determination values that were significant at *p*-level of 0.05. All data were presented as mean ± SEM. Statistical significance was defined as *p* ≤ 0.05.

## Results

### Effect of Olanzapine on Body Weight

**FIGURE 1:**
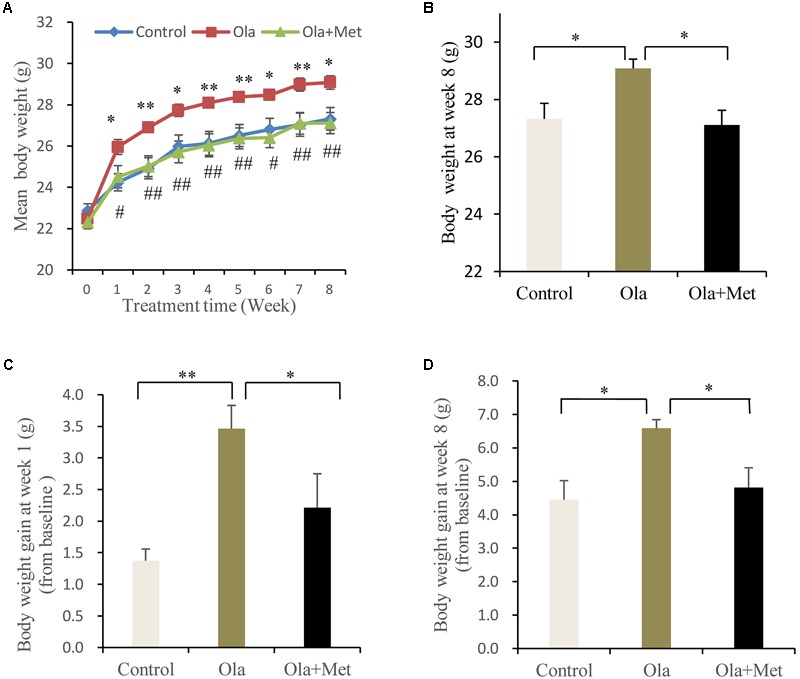
Comparison of the body weight between different treatment groups in C57BL/6 mice. C57BL/6 mice were treated with olanzapine (4 mg/kg/day, Ola), olanzapine (4 mg/kg/day, Ola) + metformin (150 mg/kg/day, Met), or saline for 8 weeks. **(A)** Body weight of mice from olanzapine group (Ola), olanzapine + metformin group (Ola +Met), and control group (Control) during 8-week of treatment. ^∗^*p* < 0.05, ^∗∗^*p* < 0.01, Ola vs. Control group; ^#^*p* < 0.05, ^##^*p* < 0.01, Ola +Met vs. Ola group. **(B)** Body weight measured at the end of 8-week treatment. **(C)** Body weight gain after the first week of treatment. **(D)** Body weight gain after 8 weeks treatment. All the results (*n* = 10 for each group) were expressed as mean ± SEM. ^∗^*p* < 0.05, ^∗∗^*p* < 0.01.

No significant difference was found in the body weight of the three groups (one-way ANOVA, *F*_2,27_ = 1.029, *p* = 0.371) prior to any treatment. However, olanzapine treatment induced a significantly higher body weight than control group and metformin group during (**Figure 1A**) and also after (**Figure 1B**, one-way ANOVA, *F*_2,27_ = 0.521, *p* = 0.012) the 8 weeks of treatment, although all three groups displayed significantly increased mean body weight after drug administration. The alteration of body weight from baseline to week 1 and week 8 was summarized in **Figures 1C,D**. As indicated in the figure, the increase of mean body weight in mice was significantly higher in olanzapine group than control group at week 1 and week 8 (one-way ANOVA, *F*_2,27_ = 7.217, *p* = 0.003; *F*_2,27_ = 5.28, *p* = 0.012), moreover, treatment with metformin plus olanzapine significantly ameliorated the mean body weight increase induced by olanzapine at week 1 and week 8 (*p* = 0.032 and *p* = 0.018, respectively). The statistic power of body weight gain at week 1 and week 8 was 0.77 and 0.67, respectively.

### Effect of Olanzapine on Fasting Glucose, Fasting Insulin, HOMA-IR, OGTT, and AUCg

**FIGURE 2:**
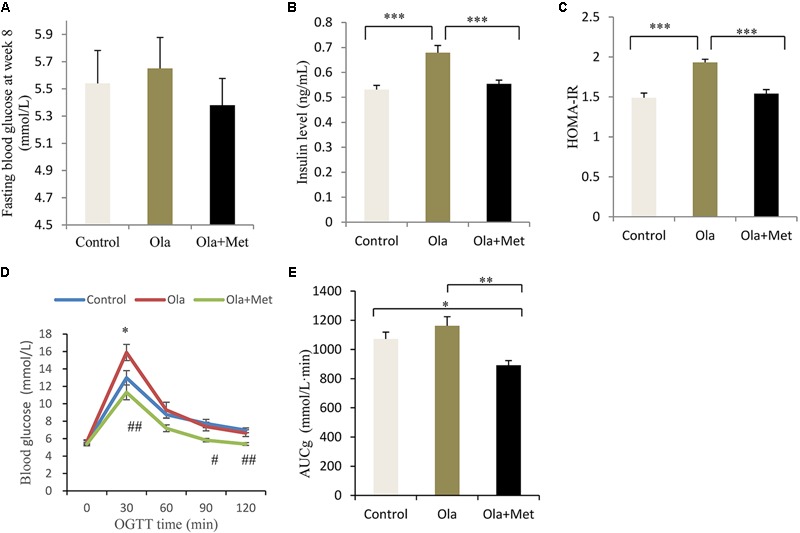
Comparison of the fasting glucose, insulin level, insulin resistance, and glucose tolerance between different treatment groups in C57BL/6 mice. C57BL/6 mice were treated with olanzapine (4 mg/kg/day, Ola), olanzapine (4 mg/kg/day, Ola) + metformin (150 mg/kg/day, Met), or saline for 8 weeks. Effect of different treatment groups on fasting glucose **(A)**, insulin level **(B)**, and HOMA-IR **(C)** at the end of 8-week treatment. **(D)** Oral glucose tolerance test (OGTT) on overnight fasted mice from olanzapine group, olanzapine + metformin group, and control group after 8 weeks of treatment. Blood glucose level was measured before and 30, 60, 90, and 120 min after glucose administration (2 g/kg body weight). ^∗^*p* < 0.05, Ola vs. Control group; ^#^*p* < 0.05, ^##^*p* < 0.01, Ola +Met vs. Ola group. **(E)** Area under curve of glucose (AUCg) of three treatment groups determined by OGTT. AUCg was calculated following trapezoidal rule from 0 to 120 min. Values (*n* = 10 for each group) were reported as mean ± SEM. ^∗^*p* < 0.05, ^∗∗^*p* < 0.01, and ^∗∗∗^*p* < 0.001.

As shown in **Figures 2A–C**, after 8 weeks of drug treatment, olanzapine-treated mice significantly increased fasting insulin level and HOMA-IR compared with control group mice (one-way ANOVA, *F*_2,27_ = 29.724, *p* < 0.001; *F*_2,27_ = 29.724, *p* < 0.001), whereas no significant difference was found in the fasting glucose between groups (one-way ANOVA, *F*_2,27_ = 0.37, *p* = 0.694). Moreover, we assessed the effect of metformin on the metabolic disturbances induced by olanzapine and found that metformin remarkably reversed olanzapine-induced fasting insulin elevation and insulin resistance (both *p* < 0.001), which was consistent with previous studies (Wang et al., 2012).

To investigate insulin resistance and pancreatic beta-cell function, we conducted the OGTT at week 8. Compared with olanzapine group, metformin plus olanzapine treated mice significantly reduced the blood glucose level at OGTT 30, 90, and 120 min (**Figures 2D,E**), and the glucose level was also lower compared with placebo group mice at OGTT 90 and 120 min. Oral glucose tolerance test following 8 weeks of treatment in the mice revealed that AUCg values were significantly lower in the metformin group compared with olanzapine group (one-way ANOVA, *F*_2,27_ = 7.787, *p* = 0.001). The AUCg value did not differ between the olanzapine treatment and control group (*p* = 0.209). The statistic power of fasting insulin level, HOMA-IR, and AUCg at the end of 8 weeks was 0.98, 0.96, and 0.79, respectively.

### Effect of Olanzapine on Blood Lipid

**FIGURE 3:**
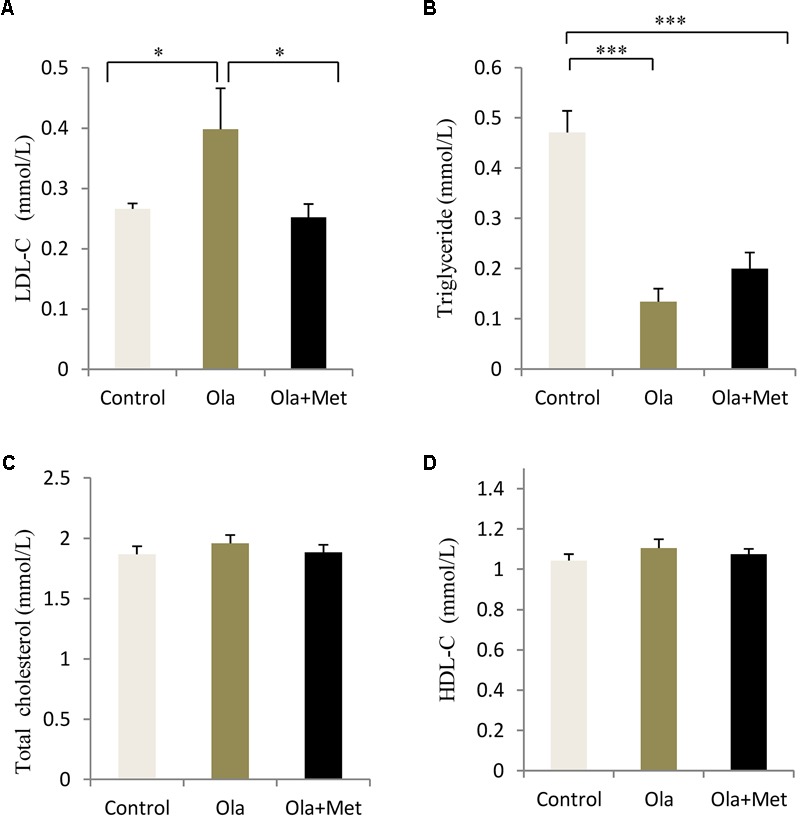
Comparison of the blood lipid between different treatment groups in C57BL/6 mice. C57BL/6 mice were treated with olanzapine (4 mg/kg/day, Ola), olanzapine (4 mg/kg/day, Ola) + metformin (150 mg/kg/day, Met), or saline for 8 weeks. Effect of different treatment groups on LDL-C **(A)**, triglyceride **(B)**, total cholesterol **(C)**, and **(D)**, HDL-C at the end of 8-week treatment. All of the results are expressed as the mean ± SEM. ^∗^*p* < 0.05, ^∗∗∗^*p* < 0.001.

In order to evaluate whether olanzapine could induce any significant difference in blood lipid between control and olanzapine treatment animals, we measured the serum total cholesterol, HDL-C, LDL-C, and triglyceride levels in the three groups after treatment completion. Olanzapine group displayed a significantly higher serum LDL-C level than control group (*p* = 0.034), which could be massively improved by metformin (**Figure 3A**, *p* = 0.02), while no significant difference was found in the level of total cholesterol and HDL-C between the treatments (one-way ANOVA, *F*_2,27_ = 0.536, *p* = 0.591; *F*_2,27_ = 0.765, *p* = 0.475). In addition, the triglyceride levels in metformin treatment group was significantly lower than the olanzapine treatment group and control group (**Figure 3**). The statistic power of LDL-C and TG at the end of 8 weeks was 0.79 and 0.97.

### Effect of Olanzapine on TCF7L2 Expression in Liver, Skeletal Muscle, Adipose, and Pancreas

**FIGURE 4:**
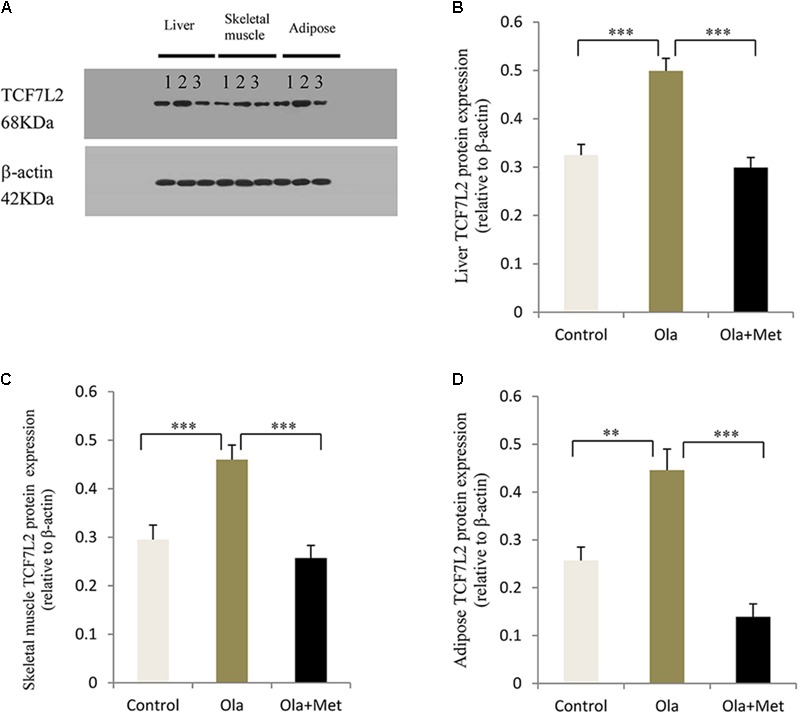
Effect of olanzapine on TCF7L2 protein expression in liver, skeletal muscle, and adipose tissue in C57BL/6 mice. C57BL/6 mice were treated with olanzapine (4 mg/kg/day, Ola), olanzapine (4 mg/kg/day, Ola) + metformin (150 mg/kg/day, Met), or saline for 8 weeks. **(A)** Protein expression level of TCF7L2 in liver, skeletal muscle, and adipose tissue of mice was measured via western blotting. Representative immunoblot images of TCF7L2 are shown. 1, 2, and 3 represents Control, Ola, and Ola + Met group, respectively. **(B)** Quantitative analysis was used to qualify the TCF7L2 protein expression level in liver. **(C)** Quantitative analysis was used to qualify the TCF7L2 protein expression level in skeletal muscle. **(D)** Quantitative analysis was used to qualify the TCF7L2 protein expression level in adipose tissue. *n* = 10 for each group. All of the results are expressed as the mean ± SEM. ^∗∗^*p* < 0.01, ^∗∗∗^*p* < 0.001.

**FIGURE 5:**
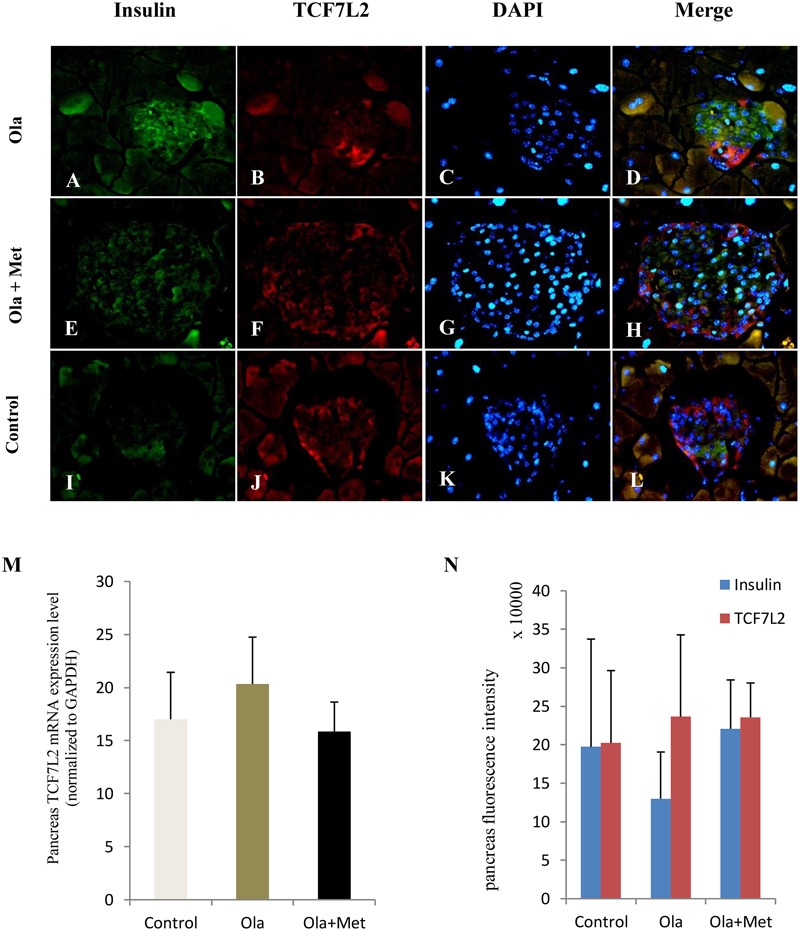
Effect of olanzapine on expressions of TCF7L2 in pancreas islets in C57BL/6 mice. C57BL/6 mice were treated with olanzapine (4 mg/kg/day, Ola), olanzapine (4 mg/kg/day, Ola) + metformin (150 mg/kg/day, Met), or saline for 8 weeks. **(A–L)** Representative images of immunofluorescence images of islets stained with antibodies to insulin (green), TCF7L2 (red), and DAPI (blue). Microscopic magnification 400×. **(M)** Quantitative analysis of *TCF7L2* mRNA expression in pancreas islets. *n* = 4 for each group. **(N)** Quantitative analysis of TCF7L2 protein expression in pancreas islets. Values are expressed as the mean ± SEM. Total pancreatic fluorescence intensity was quantified using the Image J 1.37c. *n* = 4 for each group.

TCF7L2 expressing level in liver, skeletal muscle, and adipose tissues is associated with glucose metabolism and insulin resistance (Boj et al., 2012; Kaminska et al., 2012; Singh et al., 2013). As shown in **Figures 4A–C**, we detected significant difference of TCF7L2 protein expression in liver, skeletal muscle, and adipose tissues between treatment groups (one-way ANOVA, *F*_2,27_ = 20.842, *F*_2,27_ = 13.345, and *F*_2,27_ = 20.149, respectively, all *p* < 0.001). Compared with the control, olanzapine treatment obviously increased TCF7L2 protein expression in liver, skeletal muscle, and adipose tissues (*p* < 0.001), which can be effectively reduced by metformin (*p* < 0.001). There was no significant difference in the level of TCF7L2 expression in these tissues between metformin treatment group and control group (*p* > 0.05). The statistic power of TCF7L2 protein in liver, skeletal muscle, and adipose tissues was 0.94, 0.88, and 0.95, respectively. To further explore the mechanisms of olanzapine in MetS, we determined the expression of *TCF7L2* mRNA and TCF7L2 protein in pancreas. However, there was no significant difference of *TCF7L2* mRNA or TCF7L2 protein expression among the three groups (*p* > 0.05) as shown in (**Figure 5**).

### Relationship Between TCF7L2 Protein Expression and Metabolic Measures

To investigate the relationship between the TCF7L2 protein expression and metabolic variables changes, we performed multiple linear regression analysis to evaluate the association of TCF7L2 protein expression and altered body weight, blood glucose, fasting insulin, HOMA-IR, and AUCg after olanzapine challenge. TCF7L2 protein expression was significantly correlated with changes in body weight, fasting insulin, HOMA-IR, and AUCg from baseline to week 8 (**Table 2**). The results demonstrated that the extent of increases in body weight, HOMA-IR, and AUCg exerted a greater influence on TCF7L2 protein expression elevation in liver, with coefficient of determination (*R*^2^) value of 0.461 (*p* < 0.001). Similarly, we used the same multivariate linear regression model to investigate the changes of these variables in skeletal muscle and adipose tissues, and found that the extent of increases in HOMA-IR and body weight had a greater impact on TCF7L2 protein expression elevation in skeletal muscle, with *R*^2^ value of 0.352 (*p =* 0.003), and the increase of insulin level contributed to major impact on TCF7L2 protein expression elevation in adipose tissues, with *R*^2^ value of 0.408 (*p* < 0.001).

**Table 2:** The correlation analysis between TCF7L2 protein expression and changes of metabolic measures.

TCF7L2 expression	Person correlation coefficient			
	Weight change	Fasting insulin change	HOMA-IR change	AUCg change
Liver	0.457^∗∗^	0.592^∗∗∗^	0.636^∗∗∗^	0.460^∗∗^
Skeletal muscle	0.459^∗∗^	0.499^∗∗^	0.503^∗∗^	0.399^∗^
Adipose	0.377^∗^	0.639^∗∗∗^	0.584^∗∗∗^	0.364^∗^

*Test statistic: multivariate linear regression analysis. ^∗^*p* < 0.05, ^∗∗^*p* < 0.01, ^∗∗∗^*p* < 0.001.*

## Discussion

The exact mechanism of olanzapine-induced metabolic disturbance remains unclear and numerous animal and post-mortem studies have demonstrated that Wnt signaling pathways are associated with schizophrenia and the intracellular mechanism of antipsychotic medications (Koros and Dorner-Ciossek, 2007; Sutton et al., 2007; Freyberg et al., 2010; Sutton and Rushlow, 2011). TCF7L2, a key effector of Wnt signaling pathway, performs important metabolic functions in several tissues, including the pancreas, liver, fats, and gut. In the present study, we explored the possible relationship between olanzapine-induced metabolic disturbance and TCF7L2 expression. We found that olanzapine could significantly increase TCF7L2 protein expression in the liver, skeletal muscle, and adipose tissues after 8 weeks of treatment, whereas metformin could remarkably reduce the TCF7L2 protein expression after olanzapine challenge. We further explored the relationship between TCF7L2 protein expression in these tissues and changes in metabolic variables. Our results demonstrated that the extent of increases in some metabolic variables (body weight, insulin resistance, AUCg, and insulin) actively influences the TCF7L2 expression in the liver, skeletal muscle, and adipose tissues.

Consistent with previous clinical and animal studies (Alvarez-Jimenez et al., 2008; Coccurello et al., 2009; Komossa et al., 2010; Kim et al., 2014), the present study confirms that olanzapine could significantly induce weight gain, insulin resistance, and impaired glucose tolerance. Although we did not observe any significant change of fasting glucose in mice with olanzapine treatment, the insulin resistance index was significantly higher after olanzapine challenge. Similarly, Girault et al. (2014) reported that chronic olanzapine treatment (5 weeks) could cause increase in insulin without blood glucose elevation. The observed olanzapine-induced insulin resistance in this study is parallel with a previous study which demonstrated the existence of hyperinsulinemia and insulin resistance independently from body weight gain and psychiatric disease through the use of olanzapine for 9 days in healthy subjects (Teff et al., 2013). These results suggest that olanzapine exerts direct effects on some insulin-sensitive tissues independent of mechanisms underpinning the metabolic abnormalities. Although no significant difference in AUCg was observed between the olanzapine and control groups, an increasing trend in blood glucose was evident in the olanzapine group at OGTT 30 min (*p* = 0.063). The greatest weight gain was observed in the first week of drug administration, which is consistent with clinical observation that the first year is critical for development of weight gain and metabolic abnormalities in the first treated episode of psychosis (Perez-Iglesias et al., 2013; Tek et al., 2015). In the present study, we failed to observe significant alterations in blood total cholesterol and HDL-C levels after treatment with olanzapine or olanzapine plus metformin. Consistently, clinical data also demonstrate that atypical APPs are associated with increased blood lipid levels in patients with schizophrenia (Pramyothin and Khaodhiar, 2010; Kaushal et al., 2012; Schreiner et al., 2012). Moreover, Koro et al. (2002) reported that olanzapine treatment is associated with a nearly fivefold increase in the prevalence of hyperlipidemia in contrast to the general population using a large database (which contains over 18,000 patients with schizophrenia). The effect of APPs on blood lipid profile in rodent models seems controversial, which showed no alteration (Albaugh et al., 2006), or significant increase in triglyceride level after chronic olanzapine administration (Skrede et al., 2012; Zugno et al., 2012). Yet, a recent animal study (Horska et al., 2016) demonstrated that olanzapine is associated with hypertriglyceridemia and lowered LDL-C levels at the 8th day of olanzapine treatment, but these alterations could not persist after 8 weeks of olanzapine administration, and no significant alteration in blood lipid profiles was detected in later phase of olanzapine treatment. In the present study, we have observed a massively decreased triglyceride after olanzapine treatment. This seems contradictive with MetS-related insulin resistance but features an impaired lipid oxidation caused by olanzapine. In line with our findings, Albaugh et al. (2012) also reported a significantly reduced triglycerides and free fatty acids after olanzapine challenge *in vivo*. They further demonstrated that this is largely due to the rapid and inappropriate utilization of lipids triggered by olanzapine. Although data from previous literature remain controversial, in our study, 4 mg/kg dose of olanzapine did not significantly elevate triglyceride levels, possibly because of the short duration of treatment or the improper dosage of olanzapine. However, our data showed that olanzapine could significantly increase LDL-C levels, which is consistent with previous reports (Kaushal et al., 2012; Shao P. et al., 2013).

Recently, metformin was shown to effectively attenuate antipsychotic-induced weight gain, insulin resistance, and glucose dysregulation (Hasnain et al., 2010; Praharaj et al., 2011; Wang et al., 2012; Chen et al., 2013; Jarskog et al., 2013; Boyda et al., 2014). Therefore, we examined the effects of metformin against olanzapine-induced metabolic abnormalities. Our findings are consistent with previous studies, that metformin could ameliorate olanzapine-induced metabolic abnormalities, such as weight gain, glucose intolerance, and insulin resistance. Meanwhile, metformin reduced TCF7L2 protein expression in liver, skeletal muscle, and adipose tissues, which is much higher in olanzapine treatment group, suggesting a close association between olanzapine-induced metabolic disturbance and TCF7L2 expression. The results were further supported by the fact that obesity surgery-induced weight loss could regulate the alternative splicing of TCF7L2 in subcutaneous fat. Moreover, the TCF7L2 variant is associated with fasting glucose as well as impaired insulin action in adipose tissue (Kaminska et al., 2012).

TCF7L2 is one of the strongest susceptibility genes for T2DM across different ethnicities (Grant et al., 2006). Among the *TCF7L2* polymorphisms-associated metabolic disturbance, the T-allele of rs7903146 in *TCF7L2* is the most consistent loci which is linked to schizophrenia and schizoaffective disorders (Hansen et al., 2011). As a component of the β-catenin/TCF transcription factor, TCF7L2 plays an important role in conveying Wnt signaling pathway in regulating gene expression. It has been suggested that Wnt signaling pathway and β-catenin/transcription factor could suppress hepatic gluconeogenesis through a liver-specific TCF7L2 dominant-negative transgenic mouse model (Ip et al., 2015). Animal studies also reported a strong association between liver-specific TCF7L2 overexpression and increased hepatic glucose production, and as an example, liver-specific TCF7L2 overexpression could increase hepatic glucose production (Boj et al., 2012). The role of TCF7L2 extends to non-pancreatic tissues, a recent study that revealed that *TCF7L2* overexpression in non-pancreatic tissues leads to worsened glucose intolerance, and that the function of TCF7L2 in maintaining glucose metabolic balance in peripheral tissues may be more robust (Bailey et al., 2015). Previous studies have shown that antipsychotic medications may exert their actions by modulating the activity and expression of Akt/GSK-3β and Wnt-related intracellular signaling factors (Freyberg et al., 2010; Sutton and Rushlow, 2011). For example, administration of haloperidol or clozapine could alter GSK-3 and β-catenin protein levels in the rat prefrontal cortex while both GSK-3 and TCF7L2 transcription factors are key downstream regulators in the canonical Wnt/β-catenin pathway (Struewing et al., 2010). Our data imply that the altered TCF7L2 expression may be related to the effect of olanzapine on metabolic tissues.

In the present study, TCF7L2 protein levels were significantly higher in the liver, skeletal muscle, and adipose tissues of the olanzapine-treated mice than that in the control, and significantly lower in metformin-plus-olanzapine-treated mice. Interestingly, TCF7L2 protein expression in the liver, skeletal muscle, and adipose tissues was positively correlated with changes in body weight, fasting insulin, HOMA-IR, and AUCg. To our knowledge, this is the first animal study to examine the association between TCF7L2 expression and olanzapine-induced metabolic abnormalities. The function of TCF7L2 in pancreas is well-studied using TCF7L2-overexpressing transgenic mice, Savic et al. (2011) have demonstrated robust glucose intolerance in multiple non-pancreatic tissues, including brain, stomach, intestine, and pancreas, and *TCF7L2-*null mice displayed improved glucose tolerance and lower insulin levels. Similarly, liver-specific knockout mice exhibit improved glucose homeostasis, and that liver-specific overexpression of *TCF7L2* mRNA leads to hepatic glucose production (Boj et al., 2012). Similarly, feeding can influence the overexpression of *TCF7L2* mRNA in epididymal fat tissue of C57BL/6J mice; moreover, high concentrations of insulin could inhibit the TCF7L2 level in adipocytes (Chen et al., 2015). Our data demonstrated that TCF7L2 expression in the liver and adipose tissue may play a critical role in regulating glucose metabolism. An explanation for the altered TCF7L2 expression in liver and adipose tissues may be related to weight gain, insulin resistance, and insulin level elevation induced by APPs. Interestingly, hyperinsulinemia could increase TCF7L2 mRNA expression, and subjects with low insulin sensitivity had higher TCF7L2 mRNA expression in skeletal muscle tissue (Karczewska-Kupczewska et al., 2016). In line with these data, we hypothesize that increased TCF7L2 expression in skeletal muscle might promote glucose uptake during insulin resistance conditions. Despite of the observed changes in TCF7L2 expression in liver, skeletal muscle, and adipose tissue, we did not use inhibitors to antagonize or suppress TCF7L2 specifically, and thus it remains uncertain whether the olanzapine-induced metabolic dysfunction is mediated by TCF7L2. Intriguingly, a study of African-American patients with schizophrenia reported an interaction between APPs treatment and TCF7L2 under a multiplicative scale (Irvin et al., 2009). Indeed, as APPs may alleviate symptoms of schizophrenia through Wnt signaling pathway mediated by D_2_ dopamine receptor (Sutton et al., 2007), our results emphasize the potential pharmacogenetical and clinical relevance of TCF7L2 for antipsychotic-induced metabolic dysfunction in schizophrenia and provide a novel mechanism of TCF7L2 in antipsychotic-induced metabolic disturbance. However, further studies are needed to determine the role of TCF7L2 and other components of Wnt signaling pathway in antipsychotic-induced metabolic disturbance.

Notably, the mRNA and protein expression levels of TCF7L2 in pancreas did not differ in different groups in our study. By contrast, previous studies reported that TCF7L2 expression in human islets increased by fivefolds in T2DM compared with nondiabetic individuals (Lyssenko et al., 2007). *In vitro*, elevated glucose concentration can reduce beta-cell proliferation and induce beta-cell apoptosis in cultured human islets, and these effects are reversible by TCF7L2 overexpression. By contrast, a previous study reported an opposite direction of regulating the level of TCF7L2 mRNA (upregulated) and protein (downregulated) in islets in diabetes (Le Bacquer et al., 2011). We observed no alterations in TCF7L2 expression in the pancreatic tissue, although the TCF7L2 is frequently considered to have physiological effects on β cells. The precise nature of the TCF7L2 expression in pancreatic and its etiological basis in APPs-induced metabolic disturbance remains the subject of future study.

Interestingly, oxidative stress also plays a key role in the higher incidence of metabolic dysfunction of schizophrenia. For example, Schiavone et al. (2017) found that redox imbalance plays a crucial role in the visceral fat elevation in an animal model of psychosis. Also, abnormal oxidative stress has been reported in first episode patients with schizophrenia, with increased level of thiobarbituric acid reactive substances and malondialdehyde, which are important end-point products of lipid peroxidation (Flatow et al., 2013). Indeed, in addition to a role in the pathophysiology of schizophrenia, oxidative stress has also been implicated in antipsychotic-induced metabolic dysfunction (Baig et al., 2010; Gilca et al., 2014). It has been reported that lipid peroxidation was altered in rat liver and brain following antipsychotic administration in rats, moreover, APPs can also elevate lipid peroxidation in human plasma (Dietrich-Muszalska et al., 2013). However, how antipsychotic work on antioxidant enzymes appears controversial and inconsistent (Parikh et al., 2003; Martins et al., 2008; Andreazza et al., 2015) in rat brain tissue after antipsychotic administration. A recent meta-analysis (Flatow et al., 2013) also revealed that there is no replicable and significant correlation between oxidative stress indexes and clinical features. Given previous studies of altered lipid peroxidation in antipsychotic-treated rats, and oxidative stress is closely related to insulin resistance (Ando and Fujita, 2009), the potential role of oxidative stress in antipsychotic-induced metabolic dysfunction should be further elucidated.

This study has several limitations. Firstly, we utilized healthy adult mice to analyze the mechanism of olanzapine-induced metabolic disturbance. The use of a mouse model for schizophrenia would be more reasonable given that schizophrenia itself may predispose individuals to T2DM. Secondly, the limited serum volume did not allow us to measure the *TCF7L2* mRNA levels in the liver, skeletal muscle, and adipose tissues, which can be a mediator of the observed outcomes. Thirdly, although significant differences in TCF7L2 protein expression were detected in the mentioned tissues, detecting the differences of TCF7L2 expression in mice brain and gut tissues is also a sensible approach, since proglucagon gene expression in brain and gut may be controlled by TCF7L2 and Wnt signaling pathway (Shao W. et al., 2013). Finally, we only observed the effects of single antipsychotic, single dose of olanzapine, and one antidiabetic drug. Because of many atypical APPs could induce metabolic dysregulation and multiple type of antidiabetic drug could treat diabetes, it is necessary to determine whether such findings with olanzapine apply to other APPs. Further studies are required to detect the difference in metabolic measures between antipsychotic-treated normal and specific-tissue knockout mice.

## Conclusion

Our study illustrates that olanzapine induces weight gain, fasting insulin elevation, glucose intolerance, and increase of TCF7L2 protein expression in liver, skeletal muscle, and adipose tissues of mice. These metabolic abnormalities and the increased TCF7L2 expression in those tissues could be effectively ameliorated by metformin. TCF7L2 overexpression in liver, skeletal muscle, and adipose tissues may represent a potential mechanism through which metabolic changes occurred following olanzapine treatment.

## Author Contributions

RL, JO, LL, and YY conducted all the experiments and collected all data. RL analyzed the data and wrote the first draft of the manuscript. JO organized the database. RW and JZ supervised the whole work. All authors contributed to manuscript revision and approved the submitted version.

## Conflict of Interest Statement

The authors declare that the research was conducted in the absence of any commercial or financial relationships that could be construed as a potential conflict of interest.

## Footnotes

1 http://rsb.info.nih.gov/ij/
